# Transcriptomic Analysis Reveals the Antioxidant and Anti-Inflammatory Mechanisms of EGCG-Zn Nanoparticles in Dextran Sulfate Sodium-Induced Colitis in Mice

**DOI:** 10.3390/antiox15080924

**Published:** 2026-07-25

**Authors:** Tingting Liu, Mohan Zhou, Yuhang Deng, Feifei Huang, Jie Feng

**Affiliations:** 1 Department of Animal Science, College of Animal Sciences, Zhejiang University, Hangzhou 310058, China; 22417001@zju.edu.cn (T.L.);; 2 Zhejiang Key Laboratory of Nutrition and Breeding for High-Quality Animal Products, Hangzhou 310058, China; 3 Key Laboratory of Animal Molecular Nutrition, Ministry of Education, Hangzhou 310058, China

**Keywords:** transition metal nanoparticles, EGCG-Zn, ulcerative colitis, oxidative stress, transcriptomic analysis, intestinal barrier

## Abstract

Ulcerative colitis (UC) is a chronic inflammatory disease characterized by persistent colonic inflammation, excessive oxidative stress, and impaired barrier function. Transition metal-based nanoparticles offer promising antioxidant platforms to address oxidative stress-related pathologies. To overcome the poor gastrointestinal stability of the potent dietary antioxidant epigallocatechin gallate (EGCG), we utilized zinc-coordinated EGCG (EGCG-Zn) nanoparticles (NPs), which function as a transition metal–phenolic network, to achieve sustained colonic release and overcome the poor gastrointestinal stability of free EGCG. The therapeutic efficacy and underlying mechanisms were evaluated in dextran sulfate sodium (DSS)-induced colitis in mice. Oral administration of EGCG-Zn NPs effectively reduced oxidative stress, suppressed pro-inflammatory cytokine production, alleviated colitis symptoms, and repaired the intestinal mucus and mechanical barriers. Mechanistically, transcriptomic analysis revealed that EGCG-Zn NPs pretreatment markedly reversed DSS-induced transcriptional alterations. Integrated K-means clustering and KEGG enrichment analyses further demonstrated that these protective effects were mediated by down-regulating inflammation-associated genes and up-regulating tight junction proteins, primarily involving the modulation of calcium signaling, T-cell differentiation, and the PI3K-Akt, Wnt, NF-κB, and TNF pathways. Collectively, these findings suggest that EGCG-Zn NPs alleviate DSS-induced colitis by mitigating inflammation, suppressing oxidative stress, and promoting epithelial barrier repair, supporting their potential as a functional nutraceutical for UC management.

## 1. Introduction

Ulcerative colitis (UC) is a chronic and relapsing inflammatory disorder of the colon, characterized by continuous mucosal inflammation and epithelial barrier dysfunction. Among the recurrent symptoms are abdominal discomfort, persistent diarrhea, hematochezia, and significant weight reduction [1]. Although the precise etiology of UC remains incompletely understood, accumulating evidence indicates that excessive immune activation, oxidative stress, disruption of epithelial tight junctions, and impairment of the intestinal mucus barrier collectively contribute to disease initiation and progression [2]. Among these pathological factors, oxidative stress plays a central role in the amplification of mucosal injury [3]. Overproduction of reactive oxygen species (ROS) not only directly damages epithelial cells and intestinal barrier components but also promotes the release of pro-inflammatory cytokines, thereby aggravating intestinal inflammation and delaying tissue repair [4]. Current therapeutic strategies for UC mainly include aminosalicylates, corticosteroids, immunosuppressants, and biological agents [5]. These treatments can effectively induce or maintain remission in some patients. Nevertheless, prolonged administration of these agents may give rise to appreciable adverse effects, including myelosuppression, hypersensitivity reactions, leukopenia, and dose-related toxicity [6]. Thus, the development of safe, orally administrable functional foods with high efficacy and non-toxicity to alleviate UC has become a key focus in current UC management [7].

EGCG, the major catechin in green tea, has attracted considerable attention owing to its potent antioxidant, anti-inflammatory, and cytoprotective activities [8,9]. Previous studies have shown that EGCG can scavenge ROS, regulate inflammatory signaling pathways, and protect intestinal epithelial cells from oxidative injury, suggesting its potential value in the prevention and treatment of inflammatory bowel disease [10]. Nevertheless, the clinical and nutritional application of EGCG is greatly restricted by its poor stability in the gastrointestinal environment, rapid degradation, limited intestinal absorption, and low bioavailability. These limitations make it difficult to maintain an effective EGCG concentration at inflamed colonic sites after oral administration.

Nanotechnology-based delivery systems provide a promising strategy to improve the gastrointestinal stability and therapeutic efficiency of bioactive polyphenols [11]. In particular, metal–phenolic networks formed through the coordination between metal ions and phenolic ligands have emerged as versatile platforms for constructing biocompatible nanoparticles [12]. These systems possess several advantages, including simple preparation, high loading efficiency, pH-responsive behavior, and the ability to protect polyphenols from premature degradation [13]. Zinc ions (Zn^2+^), an essential trace element, are also closely associated with intestinal homeostasis, epithelial barrier maintenance, and immune regulation [14]. Therefore, coordination assembly between EGCG and Zn^2+^ may not only enhance the delivery performance of EGCG but also provide additional benefits for intestinal barrier repair.

In the present study, EGCG-Zn NPs based on metal–phenolic coordination were constructed to achieve sustained colonic release and improve the therapeutic efficacy of EGCG against UC. The protective effects of EGCG-Zn NPs were systematically evaluated in a DSS-induced mouse model of colitis. Particular attention was paid to their effects on clinical symptoms, colonic inflammation, oxidative stress, mucus barrier restoration, and epithelial mechanical barrier integrity [15]. Furthermore, we investigated the therapeutic mechanisms of EGCG-Zn NPs in mice with DSS-induced colitis using transcriptomic techniques. Collectively, this study elucidates that EGCG-Zn NPs mitigate experimental colitis primarily through a synergistic mechanism of suppressing oxidative stress, modulating inflammatory signaling, and promoting epithelial barrier repair. These findings provide new insights into the application of EGCG-Zn NPs as a functional nutraceutical strategy for clinical UC management.

## 2. Materials and Methods

### 2.1. Synthesis of EGCG-Zn

The synthesis of EGCG-Zn NPs was conducted in our lab, following the procedure in [16]. Full synthetic details and characterization are provided in the Supplementary Information.

### 2.2. Animal Experiments

All animal procedures were carried out with authorization of the Animal Ethical and Welfare Committee of Zhejiang University (Approval No. ZJU20260435). Male C57BL/6 mice aged 8 to 10 weeks were purchased from the Laboratory Animal Center of ZJU (Hangzhou, China). The sample size was set at *n* = 8 using power analysis based on pilot data. Mice in the control group received regular drinking water throughout the experiment. Mice in the DSS group received 2.5% dextran sodium sulfate (DSS, 36–50 kDa; MP Bio, Santa Ana, CA, USA) supplemented in the drinking water for 7 consecutive days [17,18]. According to the administered dose, the EGCG-Zn treatment groups were designated as EGCG-Zn_L_ (low-dose EGCG-Zn), EGCG-Zn_M_ (medium-dose EGCG-Zn), and EGCG-Zn_H_ (high-dose EGCG-Zn). EGCG-Zn was pre-administered at doses of 0.1 g/kg (EGCG-Zn_L_), 0.2 g/kg (EGCG-Zn_M_), and 0.4 g/kg (EGCG-Zn_H_) by daily intragastric gavage that was started 2 weeks before being subjected to DSS-induced colitis and was sustained until the end of the experiment. 5-aminosalicylic acid (5-ASA) was used as a positive control. Mice in the 5-ASA group received 2.5% DSS in the drinking water and were administered 0.1 g/kg 5-ASA by oral gavage for 7 consecutive days. The DSS concentration and the doses of the EGCG-Zn complex were selected based on previously established DSS-induced colitis models and preliminary dose-optimization experiments.

At the experimental endpoint, the mice were deeply anesthetized with 100 mg/kg pentobarbital sodium intraperitoneally before euthanasia. All mice were maintained in a specific-pathogen-free environment, alongside unrestricted access to standard chow and hydration.

### 2.3. Mouse Body Weight, Disease Activity Index, and Colon Length

Daily body weight measurements were taken for all mice during dosing, alongside simultaneous examinations of stool firmness and hemorrhagic signs. To accurately capture the overall clinical severity of the experimental colitis, these daily parameters were aggregated into the disease activity index (DAI) scoring system. The calculation formula is DAI = (Body Weight Loss Score + Fecal Consistency Score + Blood in Feces Score)/3 [19]. The colon was photographed and its length was measured to assess the degree of inflammatory response.

### 2.4. Enzyme-Linked Immunosorbent Assay (ELISA)

Following euthanasia, blood was drawn via the retro-orbital sinus and centrifuged to isolate the serum, which was subsequently preserved at −80 °C. The serum levels of specific cytokines including TNF-α, interleukin-17A (IL-17A), interleukin-6 (IL-6), interleukin-1β (IL-1β), interleukin-10 (IL-10), IFN-γ, and transforming growth factor-β (TGF-β) were then quantified utilizing standardized commercial assay kits in strict adherence to the provided protocols.

### 2.5. Histological Evaluation

To preserve architectural integrity, excised tissues were fixed in 4% paraformaldehyde, sequentially dehydrated, cleared in xylene, and embedded in paraffin wax. The resulting blocks were sectioned at a thickness of 5–10 µm and mounted onto glass slides. Prior to staining, paraffin sections underwent standard deparaffinization and rehydration, whereas frozen sections were simply rinsed with PBS. Specimens were then subjected to either Hematoxylin and Eosin (H&E) or Alcian Blue/Periodic Acid−Schiff (AB-PAS) staining. Following coverslipping, cellular morphologies and microscopic structural alterations were evaluated under a light microscope. Histopathological changes were evaluated in a blinded manner according to the Dieleman scoring system [20]. The histological score was determined based on the severity of inflammatory cell infiltration, epithelial damage, and crypt destruction, and the extent of tissue involvement. The total histological score was calculated as the sum of the individual parameter scores, with higher scores indicating more severe colonic injury.

### 2.6. Quantification of Relative Intestinal mRNA Expression of Mice

To quantify the transcriptional levels of Occludin, ZO-1, and Claudin, total RNA was isolated from colon samples using the RNA pure Total RNA kit (Thermo Fisher Scientific, Waltham, MA, USA). Subsequently, complementary DNA was synthesized utilizing the PrimeScript™ RT reagent Kit equipped with gDNA Eraser RR047A (Sevier Biotechnology Co., Ltd., Wuhan, China). Real-time qPCR assays were executed on an Applied Biosystems platform (Foster City, CA, USA). The data were analyzed following the 2−∆∆Ct method and calculated using β-actin as the normalization control. The specific primer sequences (detailed in Table 1) were commercially provided by Sevier Biotechnology (Wuhan, China).

### 2.7. Tissue Immunofluorescence

For immunofluorescence evaluation, colon sections were processed through appropriate deparaffinization, rehydration, and heat-induced antigen retrieval. To prevent non-specific binding, the samples were blocked with 10% goat serum at room temperature for a minimum of 90 min. Sections were then incubated overnight at 4 °C with primary antibodies. Following subsequent washing steps, the tissues were exposed to FITC-conjugated anti-rabbit secondary antibodies for 1 h at 37 °C and counterstained with DAPI for 2 min. Image acquisition was conducted utilizing a confocal fluorescence microscope, and the relative fluorescence intensities were quantified via Image J software (v.1.53k).

### 2.8. Transcriptome Data Processing and Analysis

RNA sequencing and data analysis were performed as detailed in the Supplementary Methods.

### 2.9. Statistical Analysis

Results are expressed as mean standard deviation (SD), derived from a minimum of three independent experiments. Intergroup differences were evaluated using one-way ANOVA, with Tukey’s post hoc test applied for multiple comparisons via SPSS 22.0. For non-normally distributed variables, specifically disease activity and histological scores, group comparisons were performed using the Kruskal–Wallis test, followed by Dunn’s multiple comparison procedure. Statistical significance was set at *p* < 0.05, and significance levels are indicated as follows: * *p* < 0.05, ** *p* < 0.01, *** *p* < 0.001.

## 3. Results

### 3.1. EGCG-Zn NPs Alleviated Disease Symptoms

**Figure 1:**
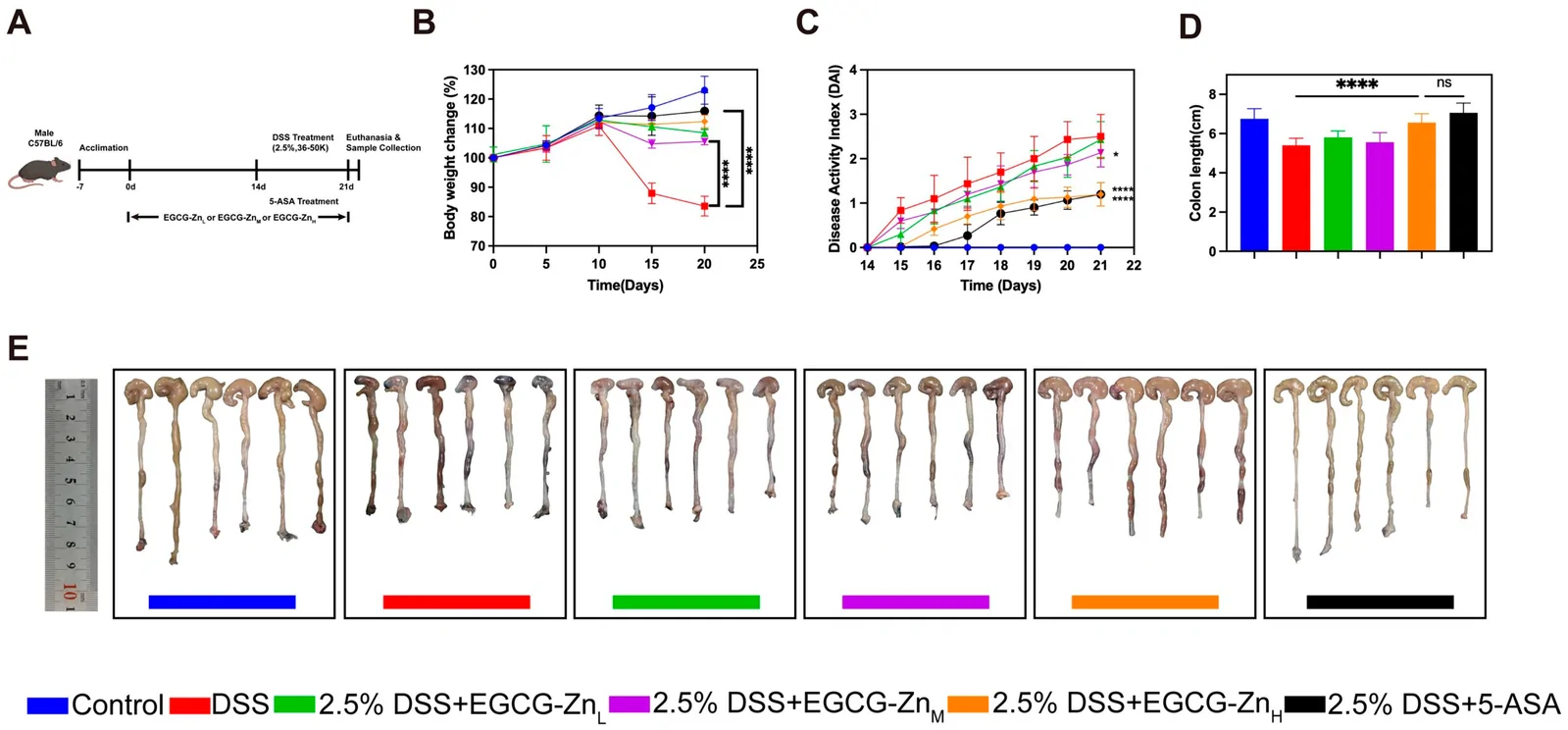
EGCG-Zn NPs relieved the clinical symptoms of colitis. (**A**) Animal experimental design. (**B**,**C**) Changes in body weight and DAI. (**D**,**E**) Representative images of the colon and colonic length. Data are presented as the mean ± SD (*n* = 8). Statistical significance: * *p* < 0.05, **** *p* < 0.0001; ns, not significant.

The successful synthesis of the EGCG-Zn NPs was verified by a series of physicochemical characterizations, with the detailed results provided in Figure S1. Having confirmed the reliable preparation of this nanoplatform, we subsequently evaluated its in vivo therapeutic efficacy using a DSS-induced acute colitis mouse model. A schematic representation of the experimental design is shown in Figure 1A. DSS-induced acute colitis manifests as diarrhea and hematochezia, accompanied by a reduction in body weight. Compared with that of control mice, the body weight of DSS-administrated mice decreased from day 3 and gradually became significant (Figure 1B). During the treatment period, the body weight and disease activity index (DAI) of mice were monitored daily. The results demonstrated that EGCG-Zn effectively mitigated DSS-induced colitis, as indicated by lower weight loss and DAI scores (Figure 1B,C). On day 21, the mice were euthanized for tissue collection and analysis. Mice in the DSS group had loose feces and bleeding conditions in the colon. In contrast, oral administration of EGCG-Zn maintained the colon length of mice at a level comparable to that of the control group, particularly in the EGCG-Zn_H_ group. The above results preliminarily illustrated that EGCG-Zn alleviated colitis symptoms, and in particular, the relief of colitis at high doses of EGCG-Zn is comparable to that of 5-ASA (Figure 1D,E). Furthermore, to evaluate the in vivo biosafety of the nanoplatform, serum biochemical parameters of the mice were analyzed. As shown in Figure S2, oral administration of EGCG-Zn NPs at different doses did not induce any obvious hepatotoxicity or nephrotoxicity, with key indicators for liver and kidney functions remaining within acceptable ranges. These results confirm the excellent biocompatibility and safety profile of EGCG-Zn NPs for in vivo applications.

### 3.2. EGCG-Zn NPs Inhibited Intestinal Inflammation and Modulated Intestinal Immunity

**Figure 2:**
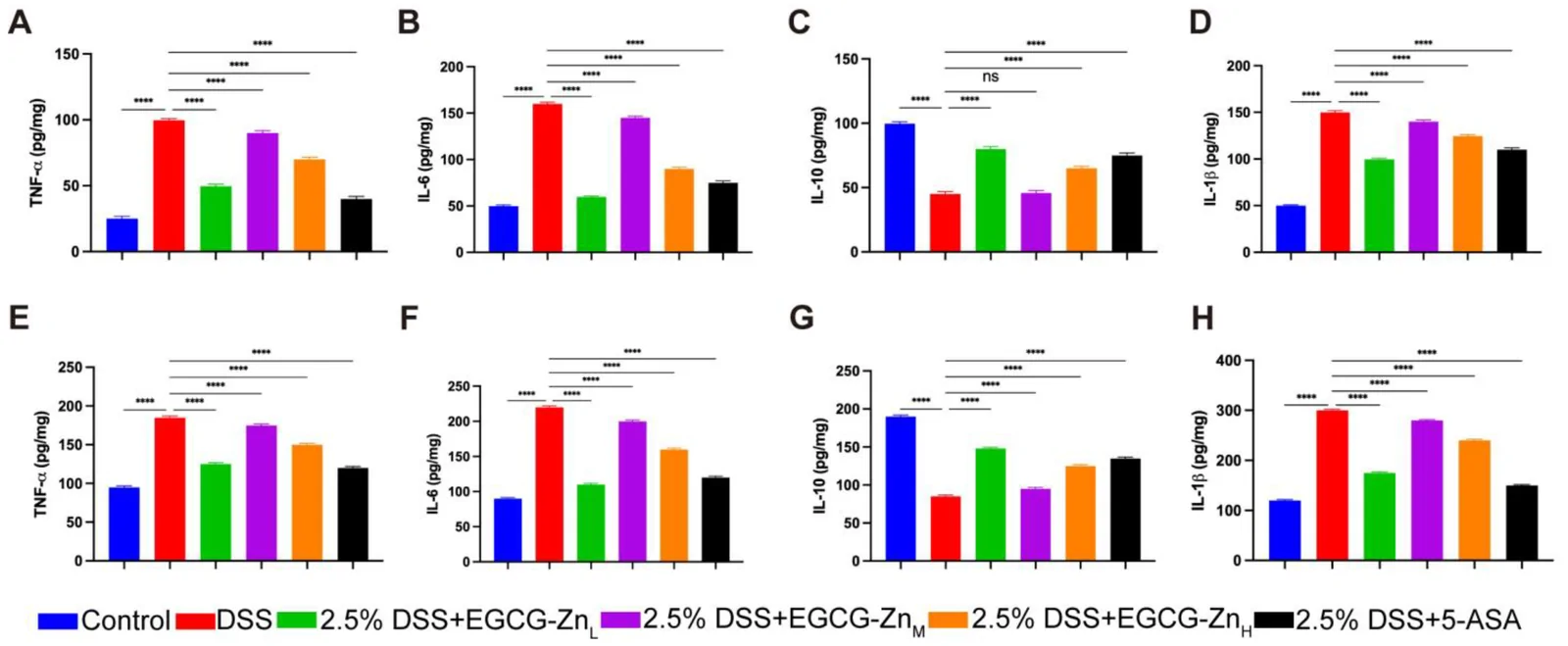
Modulation of inflammation by EGCG-Zn NPs in DSS-induced colitis mice. (**A**–**D**) Concentrations of TNF-α (**A**), IL-6 (**B**), IL-10 (**C**), and IL-1β (**D**) in colon tissue. (**E**–**H**) Concentrations of TNF-α (**E**), IL-6 (**F**), IL-10 (**G**), and IL-1β (**H**) in serum. Data are presented as the mean ± SD (*n* = 8). Statistical significance: **** *p* < 0.0001; ns, not significant.

There is mounting evidence implicating aberrations of the intestinal immune response as key drivers of both colitis development and disease exacerbation [21]. After homogenizing the serum and colon tissue, we utilized ELISA kits to measure the levels of relevant cytokines TNF-α, interleukin-6 (IL-6), interleukin-10 (IL-10) and interleukin-1β (IL-1β) within colon tissues (Figure 2A–D) and the serum (Figure 2E–H). Although TNF-α, IL-6 and IL-1β were over-expressed in the colonic mucosa of murine colitis models, EGCG-Zn treatment effectively ameliorated the disturbance in inflammatory mediator secretion.

### 3.3. EGCG-Zn NPs Mitigated Oxidative Stress Damage

**Figure 3:**
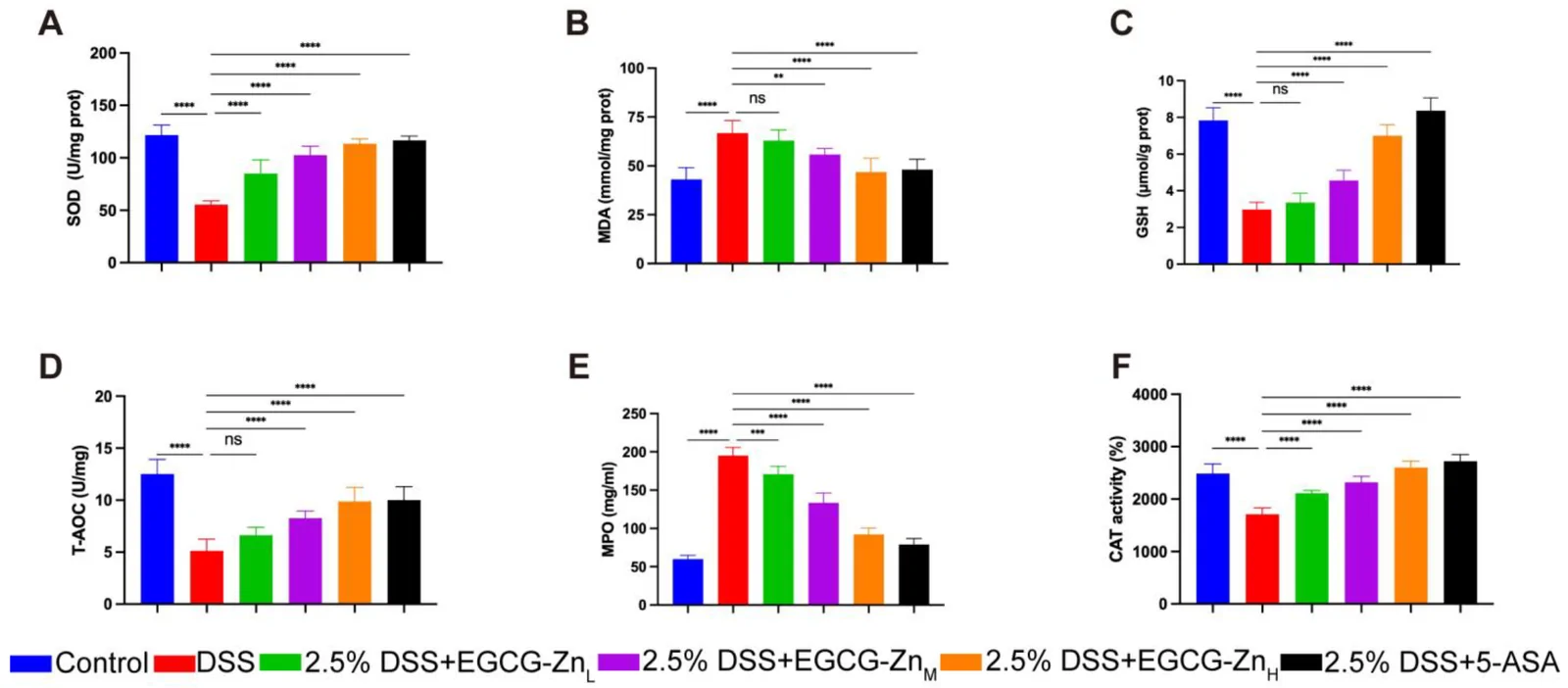
Modulation of oxidative stress by EGCG-Zn NPs in DSS-induced colitis mice. (**A**–**F**) Concentrations of SOD (**A**), MDA (**B**), GSH (**C**), T-AOC (**D**), MPO (**E**), and CAT (**F**) in serum. Data are presented as the mean ± SD (*n* = 8). Statistical significance: ** *p* < 0.01, *** *p* < 0.001, **** *p* < 0.0001; ns, not significant.

The over-activation of colonic macrophages results in abundant ROS production, thereby triggering a state of oxidative stress that manifests as lipid peroxidation, protein carbonylation, and oxidative damage to DNA [22]. The degree of oxidative stress is typically assessed by measuring the levels of SOD, MDA, GSH, T-AOC, and CAT (Figure 3A–F). EGCG-Zn treatment effectively prevented the decline in SOD, T-AOC and CAT activities in DSS-challenged colitic mice, thereby alleviating the oxidative injury triggered by inflammation (Figure 3A,D,F). The EGCG-Zn_M_ and EGCG-Zn_H_ groups showed some degree of alleviation, with the best alleviation effect in the EGCG-Zn_H_ group (*p* < 0.05). As demonstrated in Figure 3B, the level of MDA was markedly elevated in the DSS group (*p* < 0.05), and there was no remarkable mitigating effect of EGCG-Zn_L_ (*p* > 0.05). MPO, a lysosomal enzyme localized in neutrophils, also serves as a reliable indicator of the degree of neutrophil infiltration into tissues [23]. DSS markedly elevated the expression of MPO (*p* < 0.05). Compared to EGCG-Zn_L_ and EGCG-Zn_M_, EGCG-Zn_H_ significantly reduced the expression of MPO (Figure 3E). These findings demonstrate that EGCG-Zn administration effectively ameliorates colonic tissue injury induced by neutrophil infiltration.

### 3.4. EGCG-Zn NPs Ameliorated Histopathological Damage and Repaired the Mucus Barrier

**Figure 4:**
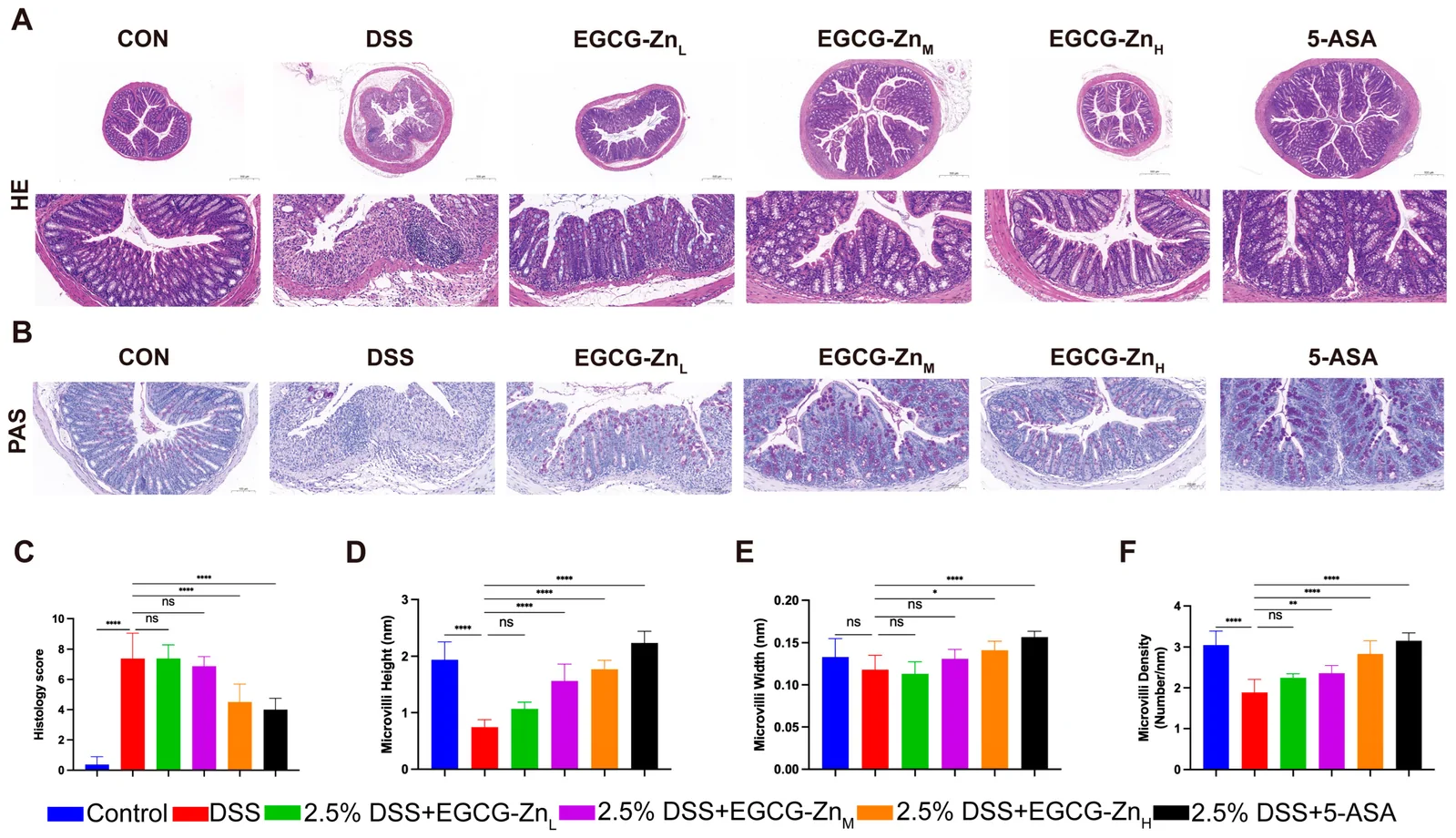
EGCG-Zn NPs ameliorated histopathological damage and repaired the mucus barrier. (**A**) Representative pictures of H&E staining. (**B**) Representative images of AB-PAS staining. (**C**) Histological scoring of colon tissues. (**D**–**F**) Quantitative analysis of villus height, width and density. Scale bars = 100 μm. Data are presented as the mean ± SD (*n* = 8). Statistical significance: * *p* < 0.05, ** *p* < 0.01, **** *p* < 0.0001; ns, not significant.

The impact of EGCG-Zn on colonic histomorphology in DSS-treated mice is illustrated in Figure 4A. As for the control group, the crypt architecture was well preserved, showing regular alignment, numerous goblet cells, and a complete absence of infiltrating inflammatory cells. In contrast, colitic mice displayed disorganized colonic crypt architecture, along with goblet cell shedding and infiltration of inflammatory cells that extended into both the mucosal and submucosal layers. Relative to the DSS-challenged cohort, mice treated with varying doses of EGCG-Zn exhibited dose-dependent reductions in colonic histopathology. Notably, the epithelial integrity and crypt architecture were remarkably preserved in both the high-dose EGCG-Zn_H_ and the 5-ASA positive control groups. Furthermore, the robust secretion of mucins by goblet cells established a thick protective mucosal layer, effectively restricting luminal antigen and bacterial translocation [24,25]. AB-PAS staining revealed that the number of mucus-containing goblet cells was markedly decreased in colitic mice, whereas EGCG-Zn_H_ treatment significantly counteracted goblet cell depletion and mucosal layer erosion (Figure 4B). Consistently, the histological score was significantly increased after DSS exposure and was reduced by EGCG-Zn treatment in a dose-dependent manner (Figure 4C). Moreover, DSS significantly decreased microvillus height, width, and density, suggesting impaired intestinal barrier ultrastructure. EGCG-Zn administration restored these microvillus parameters, with the high-dose group showing the most pronounced protective effect (Figure 4D–F). These results demonstrate that EGCG-Zn nanoparticles effectively alleviate DSS-induced colonic injury and enhance intestinal barrier integrity by preserving mucosal architecture, mucus secretion, and microvillus morphology.

### 3.5. EGCG-Zn NPs Enhanced Intestinal Epithelial Barrier Function

**Figure 5:**
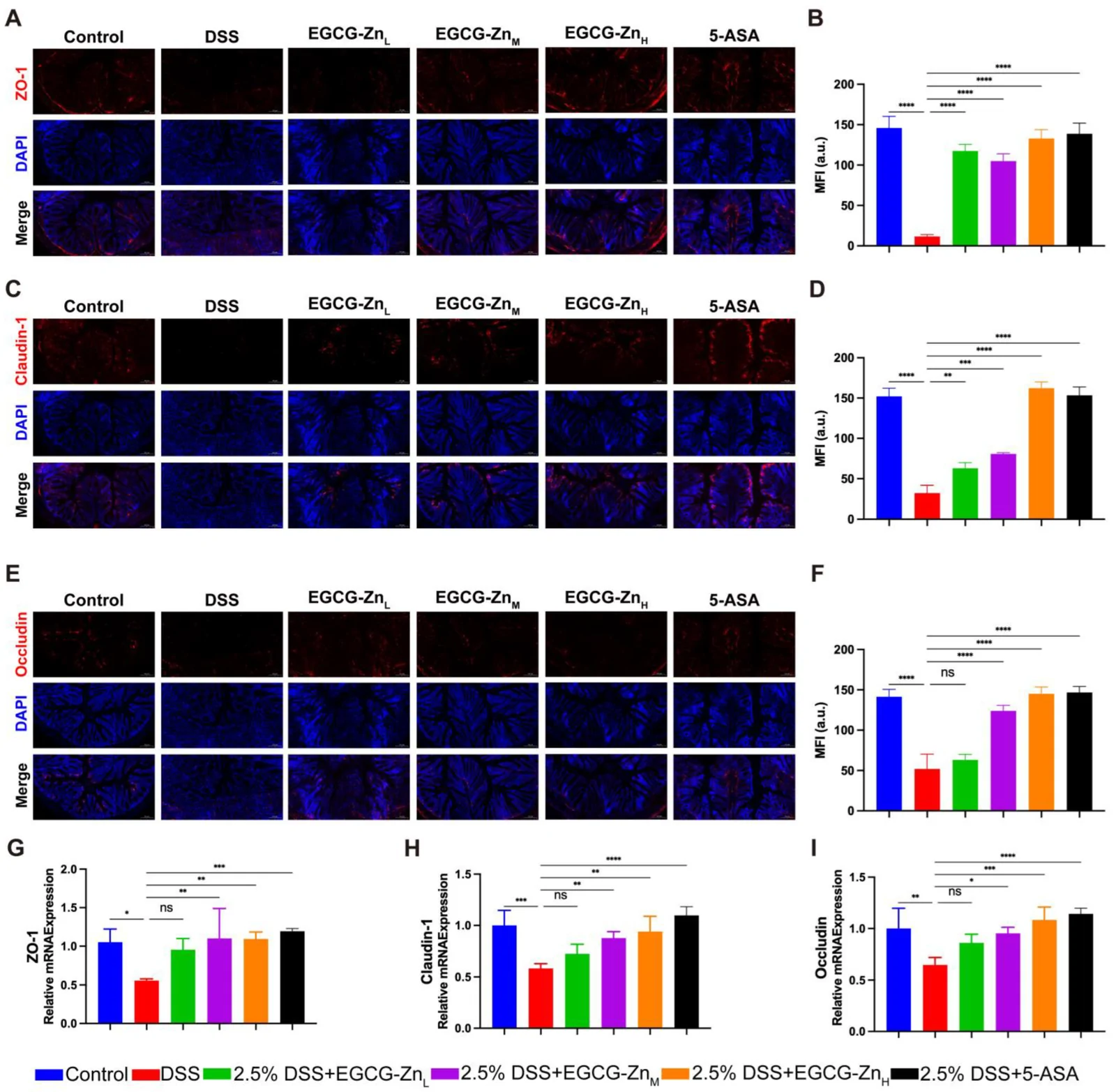
EGCG-Zn NPs enhanced intestinal epithelial barrier function. (**A**,**C**,**E**) Representative immunofluorescence staining of tight junction proteins ZO-1, Claudin-1, and Occludin in colon tissues (**B**,**D**,**F**). The relative fluorescence intensity of ZO-1, Claudin-1 and Occludin. (**G**–**I**) The relative mRNA expression levels of intestinal tight junction-related genes in the colon. Scale bars = 100 μm. Data are presented as the mean ± SD (*n* = 8). Statistical significance: * *p* < 0.05, ** *p* < 0.01, *** *p* < 0.001, **** *p* < 0.0001; ns, not significant.

**Figure 6:**
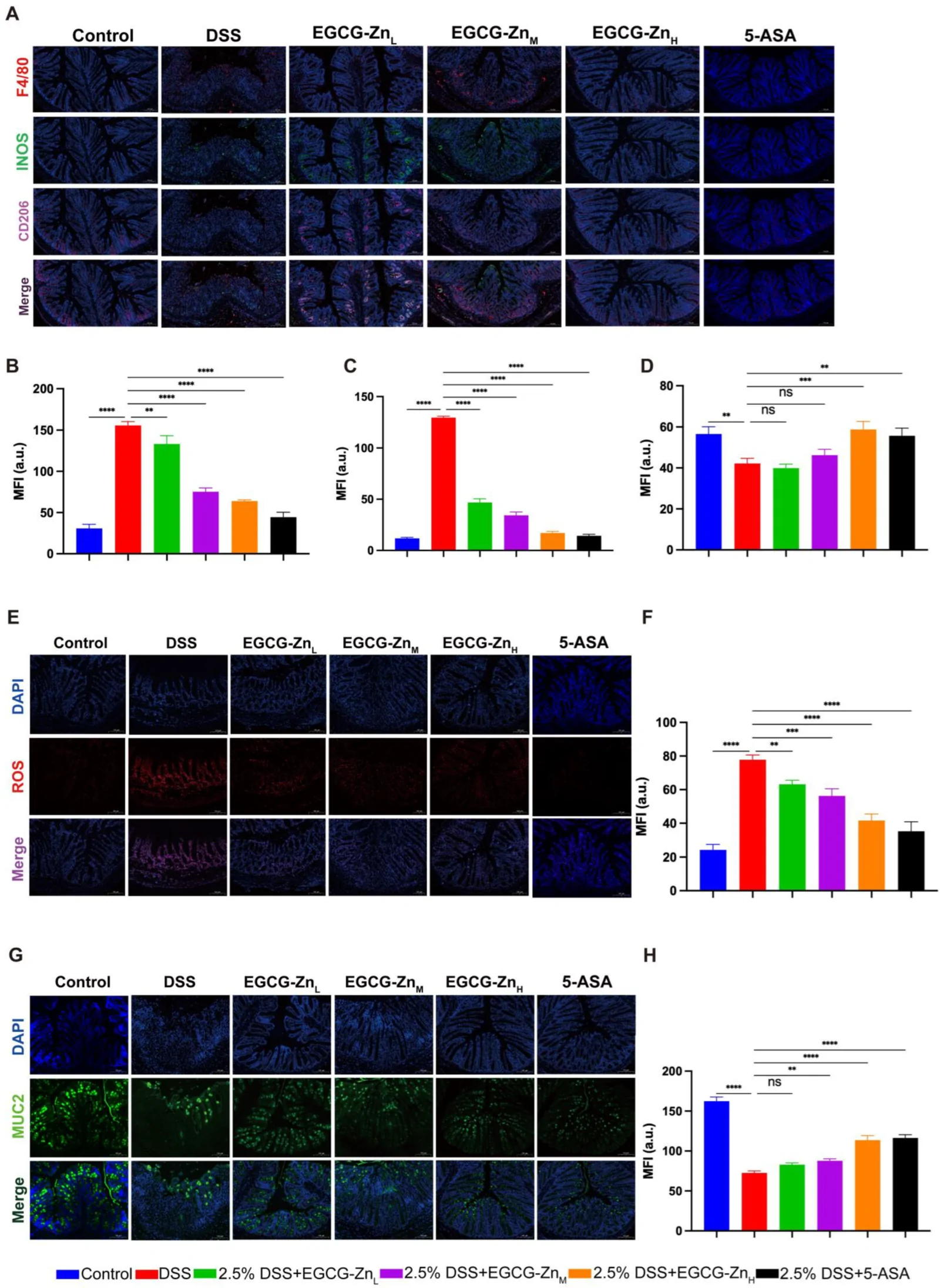
Restoration of the colonic mucosa barrier and anti-inflammatory activity of EGCG-Zn NPs for colitis therapy in mice. (**A**) Immunofluorescence staining images of F4/80 (red), INOS (green) and CD206 (pink) in colon tissues of each group. (**B**–**D**) The corresponding immunofluorescence intensity of F4/80, INOS and CD206 in colon tissue. (**E**) Immunofluorescence staining images of ROS (red) in colon tissues of each group. (**F**) The corresponding immunofluorescence intensity of ROS in colon tissue. (**G**) Immunofluorescence staining images of MUC2 (green) in colon tissues of each group. (**H**) The corresponding immunofluorescence intensity of MUC2 in colon tissue. Data are presented as the mean ± SD (*n* = 8). Statistical significance: ** *p* < 0.01, *** *p* < 0.001, **** *p* < 0.0001; ns, not significant.

ZO-1, Claudin-1, and Occludin are representative tight junction (TJ)-associated proteins that are essential for maintaining intestinal epithelial barrier integrity [26,27]. Claudin-1 and Occludin are integral transmembrane proteins that constitute the structural backbone of tight junction strands and regulate paracellular permeability between adjacent intestinal epithelial cells [28]. ZO-1, a cytoplasmic scaffolding protein, links these transmembrane TJ proteins to the perijunctional actin cytoskeleton, thereby stabilizing the TJ complex and preserving epithelial polarity and barrier function [29]. Disruption or down-regulation of ZO-1, Claudin-1, and Occludin compromises TJ architecture, increases epithelial permeability, and facilitates luminal antigen and bacterial translocation, ultimately aggravating intestinal inflammation [30]. Therefore, restoration of these TJ proteins indicates improved intestinal epithelial barrier integrity and contributes to the attenuation of DSS-induced colitis. Immunofluorescence staining was performed to detect ZO-1, Claudin-1, and Occludin, thereby evaluating the impact of EGCG-Zn on the intestinal epithelial barrier. In mice exposed to DSS in drinking water, the expression of these three tight junction proteins was markedly reduced (*p* < 0.05), indicative of epithelial barrier structural impairment (Figure 5A–F). In contrast, EGCG-Zn treatment effectively prevented the reduction in all three tight junction proteins (*p* < 0.05), thereby preserving intestinal permeability and safeguarding the mechanical barrier function. To further evaluate intestinal barrier integrity, the mRNA expression of tight junction proteins was quantified in intestinal tissues from mice (Figure 5G–I). Compared with the control group, DSS group significantly down-regulated the mRNA expression of tight junction proteins ZO-1, Claudin-1, and Occludin (*p* < 0.05), while EGCG-Zn_H_ effectively alleviated the decrease in mRNA expression of these two proteins, with a better effect than EGCG-Zn_L_ and EGCG-Zn_M_. Furthermore, to determine whether EGCG-Zn promotes macrophage polarization to restore immune homeostasis and exert anti-inflammatory effects, we performed immunofluorescence staining for F4/80 and iNOS as well as CD206. F4/80 was used as a general marker of murine macrophages, whereas CD206 and iNOS were used as markers associated with M2-like anti-inflammatory and M1-like pro-inflammatory macrophages. The results revealed that EGCG-Zn treatment showed a declining trend in M1 macrophages and an increasing trend in M2 macrophages in inflamed colons, indicating a shift in macrophage phenotype from M1 to M2 (Figure 6A–D). Compared with the DSS group, intestinal damage caused by DSS was alleviated and colonic ROS content was reduced following EGCG-Zn administration (Figure 6E,F). The results showed that treatment with EGCG-Zn effectively alleviated the oxidative stress injury of colon induced by DSS, and the effect was more effective than that of EGCG-Zn_L_ and EGCG-Zn_M_ groups. The result showed the high expression of the MUC2 protein in the control group, which is the major component of intestinal mucus structures. Immunofluorescence staining demonstrated a significant down-regulation of MUC2 expression in the colonic tissues of the DSS group compared with the control group, indicating a substantial impairment of the colonic mucus barrier. Notably, EGCG-Zn nanoparticle treatment dose-dependently attenuated this DSS-induced reduction in MUC2 expression. The EGCG-Zn_H_ group exhibited the most prominent restorative effect, with MUC2 fluorescence intensity reaching levels comparable to those observed in the 5-ASA positive control group. Collectively, these results suggest that EGCG-Zn nanoparticles protect against DSS-induced mucus barrier dysfunction by up-regulating colonic MUC2 expression (Figure 6G,H).

### 3.6. Therapeutic Mechanisms of EGCG-Zn NPs on IBD

**Figure 7:**
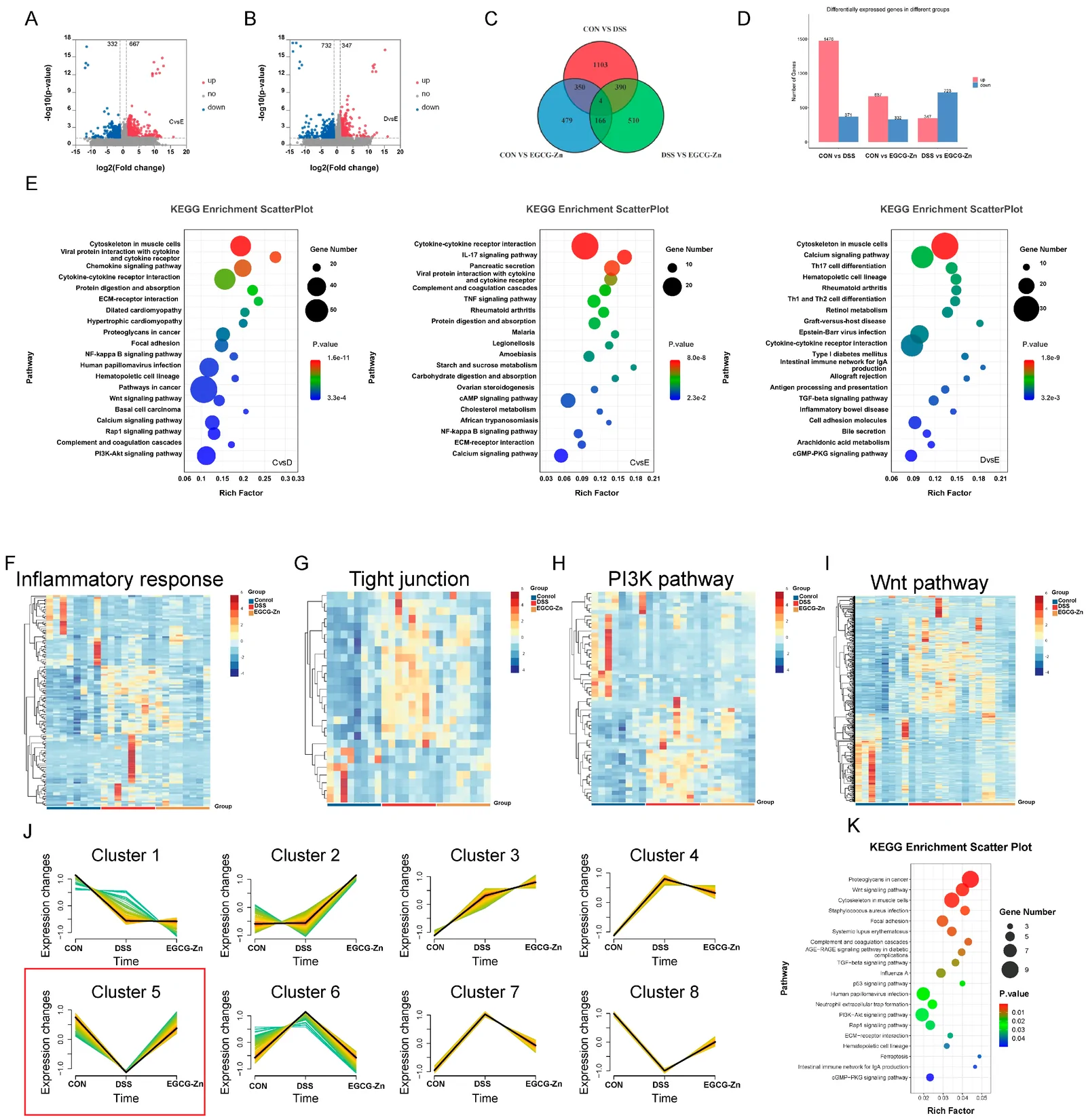
Transcriptomic analysis reveals that EGCG-Zn modulates inflammation signaling-related pathways in DSS-induced colitis. (**A**,**B**) Volcano plots showing differentially expressed genes (DEGs) between CON and DSS (**A**) and DSS and EGCG-Zn (**B**). Red dots indicate up-regulated genes, blue dots indicate down-regulated genes, and gray dots represent non-significant genes. (**C**) Venn diagram showing the overlap of DEGs among different comparison groups. (**D**) Summary of up-regulated and down-regulated genes across different comparisons. (**E**) KEGG enrichment analysis of DEGs for CON vs. DSS, DSS vs. EGCG-Zn, and CON vs. EGCG-Zn comparisons, presented as bubble plots. Bubble size represents gene number, and color indicates statistical significance. (**F**–**I**) Heatmaps showing expression patterns of genes associated with inflammatory response (**F**), tight junctions (**G**), PI3K signaling pathway (**H**), and Wnt signaling pathway (**I**) across different groups. (**J**) Cluster analysis of gene expression trends across groups, identifying representative expression patterns (Clusters 1–8). (**K**) KEGG enrichment analysis of clustered genes, highlighting key biological pathways involved in inflammation regulation, epithelial barrier function, and signaling pathways. Data are presented as mean ± SD (*n* = 8). Differential expression was determined using standard thresholds (|log2 fold change| > 1 and adjusted *p* < 0.05).

To further illustrate the therapeutic mechanism of EGCG-Zn in DSS-induced colitis, transcriptome analysis of colonic tissues was conducted at the end of pretreatment. Based on its overall protective efficacy, colon tissues from the DSS group, the EGCG-Zn_H_ group, and the control group were selected for transcriptomic analysis. As shown in Figure 7A, compared to the healthy mice, 667 up-regulated genes and 332 down-regulated genes in EGCG-Zn pretreatment DSS-induced colitis mice were detected. Meanwhile, compared to the DSS-induced colitis mice, 347 genes were up-regulated and 723 genes were down-regulated after the regulation of EGCG-Zn (Figure 7B). Venn diagrams illustrated the distinct transcriptome profiles of the control, DSS-induced colitis, and EGCG-Zn NPs-treated groups (Figure 7C,D).

We performed KEGG enrichment analysis on the DEGs to identify the pathways through which EGCG-Zn NPs exerted their immunomodulatory effects. The analysis revealed that the calcium signaling pathway, Th17 cell differentiation, Th1 and Th2 cell differentiation, and cytokine–cytokine receptor interaction were significantly enriched, suggesting that those signaling pathways are highly associated with the therapeutic mechanisms of EGCG-Zn (Figure 7E). These findings suggest that EGCG-Zn NPs exert therapeutic effects primarily through modulation of immune responses.

Furthermore, compared to healthy mice, the normalized heatmap revealed that the genes related to inflammatory response were highly expressed in DSS-induced colitis, which were evidently decreased after EGCG-Zn NPs pretreatment (Figure 7F). Similarly, EGCG-Zn pretreatment decreased the expression level of the genes related to tight junctions in DSS-induced colitis mice, indicating improved barrier function (Figure 7G). Furthermore, pathway analysis revealed the activation of PI3K-Akt and Wnt signaling pathways in the EGCG-Zn group, both of which could suppress excessive immune responses, restore immune homeostasis, and promote the repair of intestinal epithelial damage (Figure 7H,I) [31]. The above evidence convincingly indicated that EGCG-Zn could effectively eliminate oxidative stress, relieve inflammation, and recover the intestinal barrier.

To further explore dynamic transcriptional changes across different treatments, K-means clustering analysis was performed to classify DEGs into distinct expression patterns (Figure 7J). This integrative strategy, combining K-means clustering with pathway enrichment, provides a more precise identification of functionally relevant gene subsets beyond conventional DEG analysis [32]. A total of eight clusters were identified, each representing a unique temporal or condition-dependent expression trend. Among these clusters, Cluster 5 exhibited a characteristic expression pattern, in which gene expression was markedly down-regulated in the DSS group compared with the control, but significantly restored following EGCG-Zn treatment. This trend suggests that genes within Cluster 5 are closely associated with DSS-induced injury and are responsive to EGCG-Zn-mediated recovery, making them potential key targets involved in the therapeutic effect. To further investigate the biological functions of these genes, KEGG pathway enrichment analysis was conducted for Cluster 5. KEGG enrichment analysis of Cluster 5 genes revealed significant enrichment in key inflammatory and survival pathways, including NF-κB, TNF, PI3K-Akt, and cytokine–cytokine receptor interaction pathways (Figure 7K). The enrichment of these pathways indicates that the genes in Cluster 5 may play critical roles in mediating inflammatory responses and cellular survival processes.

## 4. Discussion

The therapeutic management of UC remains a significant clinical challenge, largely due to its multifactorial pathophysiology, which encompasses persistent oxidative injury, dysregulated inflammatory cascades, and compromised epithelial barrier integrity [33]. Naturally derived polyphenols, particularly EGCG, have garnered considerable interest for their potent antioxidant and anti-inflammatory properties. However, their translational potential is severely hampered by poor physicochemical stability and limited oral bioavailability [34]. To address these hurdles, we previously engineered EGCG-Zn NPs utilizing a metal–phenolic coordination network. In the current study, we demonstrate that this nanoformulation serves as an efficacious oral delivery platform, effectively shielding EGCG from premature degradation in the upper gastrointestinal tract and facilitating its localized release within the colonic milieu. Previous studies have shown that EGCG-Zn NPs are relatively stable at acidic pH. Given that zinc coordination significantly improves EGCG stability, it is reasonable to expect that the EGCG-Zn complex would maintain its stability advantage over free EGCG in the gastrointestinal tract [35,36,37]. Oral administration of EGCG-Zn NPs significantly ameliorated DSS-induced colitis in mice, as evidenced by mitigated disease activity, suppressed inflammatory infiltration, restoration of redox equilibrium, and improved mucosal barrier function.

The enhanced therapeutic outcome observed with EGCG-Zn NPs is intrinsically linked to the distinctive attributes of the metal–phenolic network [38]. Free EGCG is highly susceptible to oxidation and pH-dependent degradation, which drastically limits the concentration reaching the colonic epithelium [39]. Coordination with zinc ions likely establishes a protective shielding effect, preventing autoxidation of the gallate moiety and enabling a sustained release profile triggered by the colonic microenvironment. This delivery behavior not only elevates the local bioavailability of EGCG at the sites of active inflammation but also minimizes systemic exposure, thereby augmenting the therapeutic window.

At the tissue level, DSS challenge induces a vicious cycle of excessive ROS production and robust pro-inflammatory cytokine release, hallmarks that define the active UC state [40]. Treatment with EGCG-Zn NPs effectively disrupted this cycle by attenuating inflammatory activation and re-establishing systemic redox homeostasis [41]. Interestingly, although EGCG-Zn_L_ produced greater improvements in several inflammatory parameters, EGCG-Zn_H_ showed superior antioxidant activity. Considering the overall efficacy, including anti-inflammatory, antioxidant, and tissue-protective effects, the EGCG-Zn_H_ treatment exhibited a more comprehensive protective profile. Therefore, we selected EGCG-Zn_H_ for subsequent mechanistic analyses.

To gain deeper mechanistic insights, we performed transcriptomic profiling, which revealed that EGCG-Zn NPs partially rectified the global transcriptional aberrations associated with colitis. Notably, the down-regulation of the NF-κB and TNF signaling pathways stands out as a critical event, providing a molecular rationale for the observed suppression of inflammatory cascades [42]. Furthermore, the transcriptomic data hinted at perturbations in immune-related gene signatures, suggesting a modulatory effect of EGCG-Zn NPs on the local immunological microenvironment, although the specific immune effector cells and their phenotypic switches necessitate future elucidation via flow cytometry or immunohistochemistry.

Beyond immunomodulation, the restoration of intestinal barrier integrity is paramount for halting the translocation of luminal antigens that perpetuate mucosal inflammation [43]. Our data demonstrate that EGCG-Zn NPs promoted the concurrent recovery of the protective mucus layer and the paracellular tight junction complex. Mechanistically, this protective effect was correlated with an up-regulation of tight junction-related genes and a significant enrichment of Wnt and PI3K-Akt signaling pathways. Given that these pathways are critical for epithelial stem cell proliferation, survival, and migratory repair [44], EGCG-Zn NPs likely exert a dual function by directly mitigating inflammatory injury while simultaneously potentiating endogenous mucosal healing programs.

A crucial aspect of this nanoformulation is the inherent bioactivity of the metal ion. Zinc, beyond being a structural coordinator, is an essential trace element recognized for its pivotal role in intestinal mucosal repair and immune regulation [45]. Thus, the comprehensive protective efficacy observed here cannot be exclusively attributed to EGCG, but rather plausibly stems from a synergistic interplay where Zn^2+^ contributes to the restoration of epithelial integrity and immune tolerance. Additionally, our gene set enrichment analysis identified alterations in calcium-related signaling cascades, suggesting that intracellular calcium dynamics, which are central to cytoskeletal remodeling and tight junction assembly, may participate in the barrier-restorative effects [46,47]. Although the immunofluorescence and transcriptomic findings provide complementary evidence for the potential immunomodulatory mechanisms of EGCG-Zn, the expression of key proteins was not further quantified by Western blotting in the present study. Future studies will focus on validating the selected proteins at the protein level and clarifying their functional roles in the protective effects of EGCG-Zn against DSS-induced colitis.

While our findings robustly support the potential utility of EGCG-Zn NPs for intestinal inflammation, the translational relevance of this study is bounded by certain limitations. Foremost, the acute DSS-induced colitis model, while standard, recapitulates primarily the acute injury phase and does not fully mirror the chronic, relapsing, and heterogeneous nature of human IBD [48,49]. Future efficacy evaluations in chronic or spontaneous colitis models would be more clinically predictive.

In summary, our study confirms that the pre-developed EGCG-Zn NPs platform effectively overcomes the oral delivery barriers of free EGCG, thereby exerting pronounced protective effects against DSS-induced colitis. By bypassing premature degradation in the upper gastrointestinal tract and achieving targeted colonic release, EGCG-Zn NPs orchestrate a multifaceted therapeutic response that modulates oxidative stress, quells inflammatory signaling and fortifies the intestinal mucosal barrier. These findings advocate for the continued development of metal–phenolic coordination-based nutraceutical delivery systems as a promising and viable strategy for the management of intestinal inflammatory disorders [50].

## 5. Conclusions

In conclusion, this study employed previously developed EGCG-Zn NPs in our laboratory, constructed through metal–phenolic coordination, as an oral delivery system to enhance the gastrointestinal stability and colonic delivery efficiency of EGCG. Oral administration of EGCG-Zn NPs significantly alleviated DSS-induced colitis in mice in a dose-dependent manner, with the high-dose regimen exhibiting the most robust therapeutic efficacy. This therapeutic effect was attributed to a synergistic mechanism involving the suppression of oxidative stress, inhibition of pro-inflammatory cytokine secretion, and restoration of both the mucus layer and epithelial barrier integrity. Transcriptomic analysis further suggested that EGCG-Zn NPs exert their protective effects by down-regulating inflammatory cascades via NF-κB and TNF signaling, while concomitantly activating epithelial repair programs through PI3K-Akt and Wnt pathways, thereby reducing pro-inflammatory gene expression and reinforcing tight junction integrity. This work provides a promising oral nutraceutical strategy for UC management and supports the further development of metal–phenolic network-based delivery systems for ulcerative colitis therapy.

## Figures and Tables

**Table 1:** Primers of target genes.

Gene	Primer Sequences (5′-3′)	Primer Length (bp)
Claudin	GAAAAATGGACGAACTGGGCTCC CCAGAACGGAGGCAGCAATCAT	150
Occludin	TGGCAAGCGATCATACCCAGAG CTGCCTGAAGTCATCCACACTC	103
ZO-1	GTTGGTACGGTGCCCTGAAAGA GCTGACAGGTAGGACAGACGAT	133
Actin	CATTGCTGACAGGATGCAGAAGG TGCTGGAAGGTGGACAGTGAGG	138

## Data Availability

The original contributions presented in this study are included in the article. Further inquiries can be directed to the corresponding author.

## Supplementary Materials

The following supporting information can be downloaded at: https://www.mdpi.com/article/10.3390/antiox15080924/s1, Figure S1: Synthesis, characterization, and antioxidant activity of EGCG-Zn nanoparticles. Figure S2: The effect of EGCG-Zn NPs on serum biochemical indicators in DSS-induced colitis mice.

## Abbreviations

The following abbreviations are used in this manuscript:

UC	Ulcerative Colitis
IBD	Inflammatory Bowel Disease
DSS	Dextran Sulfate Sodium
DAI	Disease Activity Index
5-ASA	5-Aminosalicylic Acid
EGCG	Epigallocatechin-3-gallate
NPs	Nanoparticles
SPF	Specific-Pathogen-Free
ROS	Reactive Oxygen Species
SOD	Superoxide Dismutase
MDA	Malondialdehyde
GSH	Glutathione
T-AOC	Total Antioxidant Capacity
CAT	Catalase
MPO	Myeloperoxidase
TNF-α	Tumor Necrosis Factor-Alpha
IL-6	Interleukin-6
IL-10	Interleukin-10
IL-1β	Interleukin-1 Beta
IL-17A	Interleukin-17A
IFN-γ	Interferon-Gamma
TGF-β	Transforming Growth Factor-Beta
MUC2	Mucin 2
ZO-1	Zonula Occludens-1
iNOS	Inducible Nitric Oxide Synthase
CD206	Cluster of Differentiation 206
F4/80	F4/80 Antigen
NF-κB	Nuclear Factor-Kappa B
PI3K-Akt	Phosphoinositide 3-Kinase
Wnt	Wingless-Related Integration Site
TNF	Tumor Necrosis Factor
H&E	Hematoxylin and Eosin
AB-PAS	Alcian Blue/Periodic Acid–Schiff
ELISA	Enzyme-Linked Immunosorbent Assay
IF	Immunofluorescence
DAPI	4′,6-Diamidino-2-Phenylindole
cDNA	Complementary DNA
qPCR	Quantitative Real-Time Polymerase Chain Reaction
mRNA	Messenger RNA
RNA-seq	RNA Sequencing
DEGs	Differentially Expressed Genes
KEGG	Kyoto Encyclopedia of Genes and Genomes
FPKM	Fragments Per Kilobase of Transcript per Million Mapped Reads
K-means	K-Means Clustering
GO	Gene Ontology
ANOVA	Analysis of Variance
SD	Standard Deviation
PBS	Phosphate-Buffered Saline
Tris	Tris(hydroxymethyl)aminomethane
