# Supplementary material for: Transcriptomic Analysis Reveals the Antioxidant and Anti-Inflammatory Mechanisms of EGCG-Zn Nanoparticles in Dextran Sulfate Sodium-Induced Colitis in Mice
Source: Antioxidants (Basel). 2026 Jul 25;15(8):924. doi: 10.3390/antiox15080924 (PMC13510162; doi:10.3390/antiox15080924)
Supplement: Supplementary file 1 [file antioxidants-15-00924-s001.zip › antioxidants-4418770-supplementary.pdf]

# Supporting Information

Transcriptomic Analysis Reveals the Antioxidant and Anti-Inflammatory Mechanisms of EGCG-Zn Nanoparticles in dextran sulfate sodium-Induced Colitis in mice

Tingting Liu [1,2], Mohan Zhou [1,3], Yuhang Deng [1,2], Feifei Huang [1,3] and Jie Feng [1,2,3,*]

[1] Department of Animal Science, College of Animal Sciences, Zhejiang University, Hangzhou 310058, China;

[2] Zhejiang Key Laboratory of Nutrition and Breeding for High-Quality Animal Products, Hangzhou 310058, China

[3] Key Laboratory of Animal Molecular Nutrition, Ministry of Education, Hangzhou 310058, China

[*] Correspondence: fengj@zju.edu.cn

## Materials and Methods

### Synthesis of EGCG-Zn NPs

We employed a water-based, ambient-condition coordination strategy for the assembly of metal-phenolic network nanoparticles[20]. EGCG (6 mg) was dissolved in 8 mL of deionized water. Subsequently, 210 mg of $ZnCl_2$ was added to the solution. Then, 120 mg of polyvinyl pyrrolidone (PVP) was dissolved in 40 mL of deionized water, and 2 mL of this solution was added. The solution was stirred at 25 °C for 12 h and then centrifuged at 12,000 rpm for 10 min to remove excess EGCG. Finally, the crude product was purified using a freeze dryer.

### Characterization

Transmission electron microscopy (TEM) was performed using a JEM-1400 Flash microscope (JEOL). The hydrodynamic diameters of the samples were measured with dynamic light scattering (DLS, Nicomp N3000). XPS measurements were carried out via X-ray photoelectron spectroscopy (XPS, Thermo Scientific Nexsa).The ultraviolet-visible (UV-Vis) absorption spectra were carried out on a Nano Drop one. The Fourier transform infrared spectrum was collected with a Tensor 27 IR

spectrometer (Bruker, Germany). Fluorescence images were obtained via fluorescence microscope (Leica, Germany).

**EGCG-Zn NPs therapeutic efficacy against a DSS-Induced Acute Colitis Model**. Mice were randomly assigned to five groups: Control, DSS+PBS, DSS+EGCG-$Zn_L$, DSS+EGCG-$Zn_M$, and DSS+ EGCG-$Zn_H$ (n = 8 per group). The control group received normal water for 10 days, while the other groups received 2.5% (w/v) DSS for the first 7 days followed by normal water for the remaining 3 days. During the experiment, mice treated with DSS were orally administered with 0.9% NaCl, EGCG-$Zn_L$ (0.1 g/kg), EGCG-$Zn_M$ (0.2 g/kg), EGCG-$Zn_H$ (0.4 g/kg), respectively. During the experiment, stool consistency, fecal blood, and body weight of the mice were recorded daily to calculate the disease activity index (DAI). Additionally, all mice were euthanized, and the colon and blood were collected for further analysis. The length of the colon was measured, and the colon tissues were divided into two parts: one for H&E staining and the other for PAS staining. The blood samples were used for ELISA analysis.

**Transcriptomics Analysis.**

RNA extraction and library construction

Total RNA was extracted with TRIzol reagent (Invitrogen, Carlsbad, CA, USA) according to the supplier's instructions. The concentration and purity of each specimen were determined using a NanoDrop ND-1000 spectrophotometer (NanoDrop, Wilmington, DE, USA), while RNA integrity was verified on a Bioanalyzer 2100 (Agilent, CA, USA) with a RIN threshold >7.0, and further

confirmed via denaturing agarose gel electrophoresis. From 1 μg of total RNA, poly(A) transcripts were enriched by two rounds of purification with Dynabeads Oligo(dT)25-61005 (Thermo Fisher, CA, USA). The enriched mRNA was then fragmented chemically with the Magnesium RNA Fragmentation Module (NEB, cat.e6150, USA) at 94 ℃ for 5-7 min. The resultant RNA fragments served as templates for first-strand cDNA synthesis using SuperScript™ II Reverse Transcriptase (Invitrogen, cat. 1896649, USA), followed by generation of U-labeled second strands with E. coli DNA polymerase I (NEB, cat.m0209), RNase H (NEB, cat.m0297), and dUTP Solution (Thermo Fisher, cat.R0133). After end-repair and A-tailing, indexed adapters (with T-overhangs) were ligated to the fragments, and size selection was performed with AMPureXP beads. The U-labeled strands were removed by heat-labile UDG enzyme (NEB, cat.m0280), and the final library was amplified via PCR under the following cycling parameters: initial denaturation at 95 ℃ for 3 min; 8 cycles of 98 ℃ for 15 s, 60 ℃ for 15 s, and 72 ℃ for 30 s; with a final extension at 72 ℃ for 5 min. The resulting cDNA libraries had an average insert size of 300 ± 50 bp. Ultimately, paired-end sequencing (2x150 bp) was conducted on an Illumina NovaSeq™ 6000 platform (LC-Bio Technology Co., Ltd., Hangzhou, China) following the recommended protocol.

Bioinformatics analysis of RNA-seq

fastp software (https://github.com/OpenGene/fastp) was used to remove the reads that contained adaptor contamination, low quality bases and undetermined bases with default parameter. Then sequence quality was also verified using fastp. We used

HISAT2 (https://ccb.jhu.edu/software/hisat2) to map reads to the reference genome of mus_musculus Ensembl_v112. The mapped reads of each sample were assembled using StringTie (https://ccb.jhu.edu/software/stringtie) with default parameters. Then, all transcriptomes from all samples were merged to reconstruct a comprehensive transcriptome using gffcompare (https://github.com/gpertea/gffcompare/). After the final transcriptome was generated, StringTie was used to estimate the expression levels of all transcripts. StringTie was used to perform expression level for mRNAs by calculating FPKM (FPKM = [total_exon_fragments / mapped_reads(millions) × exon_length(kB)]). The differentially expressed mRNAs were selected with fold change > 2 or fold change < 0.5 and with parametric F-test comparing nested linear models (p value < 0.05) by R package edgeR (https://bioconductor.org/packages/release/bioc/html/edgeR.html).

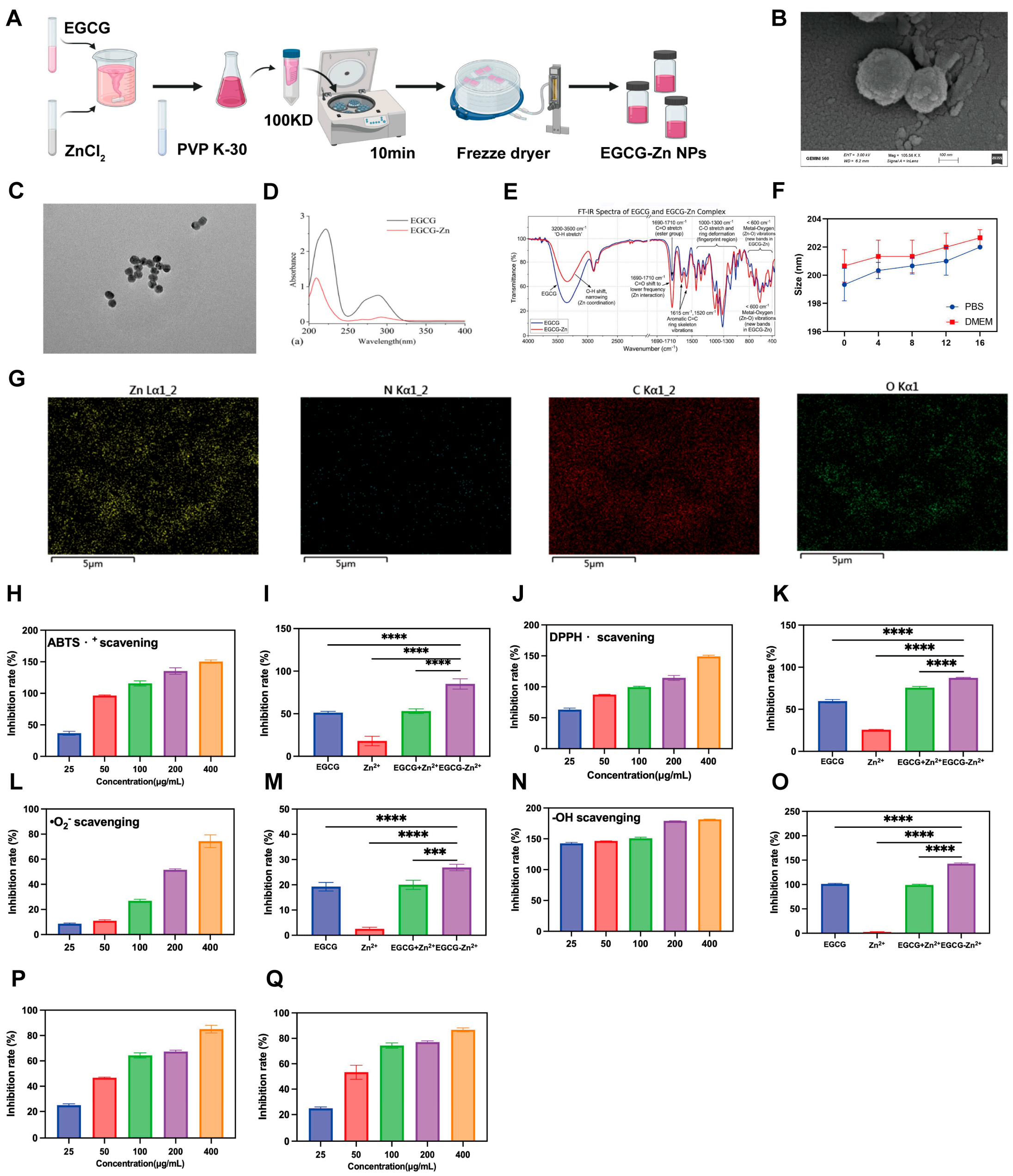


**Figure S1.** Synthesis, characterization, and antioxidant activity of EGCG-Zn nanoparticles.

(A) Schematic illustration of the preparation process of EGCG-Zn nanoparticles (EGCG-Zn NPs), including coordination between EGCG and $Zn^{2+}$ followed by PVP modification.

(B) Scanning electron microscopy (SEM) image showing the morphology of EGCG-Zn NPs.

(C) Transmission electron microscopy (TEM) image of EGCG-Zn NPs, indicating ultrasmall and well-dispersed nanostructures.

(D) UV-vis absorption spectra of EGCG and EGCG-Zn, confirming the formation of coordination complexes.

(E) FT-IR spectra of EGCG and EGCG-Zn, demonstrating the involvement of phenolic hydroxyl groups in metal coordination.

(F) Hydrodynamic size stability of EGCG-Zn NPs in PBS and DMEM over time.

(G) Elemental mapping images (Zn, N, C, and O) of EGCG-Zn NPs, indicating uniform elemental distribution.

(H-O) In vitro antioxidant activities of EGCG-Zn NPs evaluated by ABTS•$^{+}$, DPPH•, $O_2^-$, and -OH radical scavenging assays at different concentrations, as well as comparison among EGCG, $Zn^{2+}$, and EGCG-Zn formulations.

(P-Q) SOD-like and CAT-like antioxidative activity of EGCG-Zn NPs

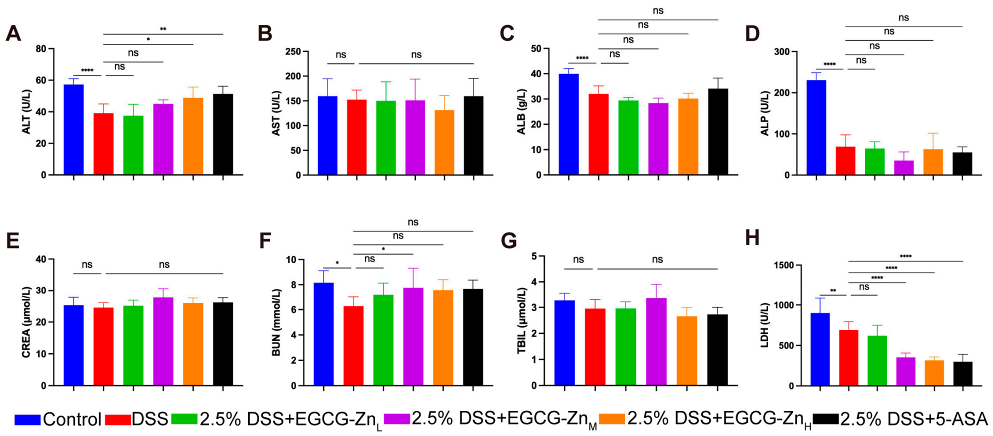


**Figure S2.** The effect of EGCG-Zn NPs on serum biochemical indicators in DSS-induced colitis mice

(A) alanine aminotransferase (ALT), (B) aspartate aminotransferase (AST), (C)

albumin (ALB), (D) alkaline phosphatase (ALP), (E) creatinine (CREA) and (F) blood urea nitrogen (BUN); (G) total bilirubin (TBIL); (H) lactate dehydrogenase (LDH). Data are presented as mean ± SD ($n = 8$). ns indicates no significant difference, *$p < 0.05$, $p < 0.01$, ***$p < 0.001$, ****$p < 0.0001$.
